# Trends in Hospitalization, Case Fatality, and Mortality for Community-Acquired Pneumonia in Portugal over 20 Years

**DOI:** 10.3390/epidemiologia7050125

**Published:** 2026-09-08

**Authors:** Rita Oliveira, Diogo Batista, Filipa Lufinha, Henrique Oliveira, António Diniz, Filipe Froes, João Gonçalves Pereira

**Affiliations:** 1 Chest Department, Unidade Local de Saúde Santa Maria, 1769-001 Lisboa, Portugal; arsampaioliveira@gmail.com (R.O.); diogobatista36@gmail.com (D.B.); adinizfirst@gmail.com (A.D.); filipe.froes@gmail.com (F.F.); 2 Department of Medicine, Unidade Local de Saúde Amadora-Sintra, 2720-276 Amadora, Portugal; filipalufinhagp@gmail.com; 3 Department of Mathematics and Centre for Mathematical Analysis, Geometry and Dynamical Systems, Instituto Superior Técnico, Lisbon University, 1049-001 Lisboa, Portugal; henrique.m.oliveira@tecnico.ulisboa.pt; 4 Emergency and Intensive Care Medicine Department, Hospital Vila Franca de Xira, 2600-009 Vila Franca de Xira, Portugal; 5 Department of Intensive Care Medicine, Faculty of Medicine, Lisbon University, 1649-028 Lisboa, Portugal

**Keywords:** community-acquired pneumonia, epidemiology, mortality, seasonality, invasive mechanical ventilation

## Abstract

Background: Community-acquired pneumonia (CAP) remains a leading cause of morbidity and mortality worldwide and a major driver of hospital admissions. Older adults are disproportionately affected, owing to immune senescence and a higher prevalence of chronic comorbidities. This study examined two decades (2000–2019) of adult hospital admissions for CAP in mainland Portugal. Methods: We analyzed adult CAP hospitalizations between 2000 and 2019 from the Portuguese Mainland Hospital Diagnosis National Database. We assessed all hospitalizations attributable to CAP over the 20 years, stratified by age group, and examined seasonal variation. Trends in hospital length of stay and in-hospital case fatality were evaluated. Results: Between 2000 and 2019, the adult CAP hospitalization rate in Portugal rose from 3.61 to 5.28 per 1000 persons-year. This was fostered by patients older than 80 years, who grew from 30.4% to 53.1% of CAP admissions. The mean age increased over time, from 70.1 ± 17.0 years to 77.7 ± 14.1 years. Hospitalization episodes of patients older than 85 years admitted for CAP increased fourfold. CAP-associated case fatality increased slightly, driven by patients older than 80 years. A modest decline was noted from 2016 to 2019 (from 23.1% to 21.8%). This was strongly correlated with mean age. Invasive mechanical ventilation was offered in a growing number of hospitalization episodes of CAP (from 3.1% in 2000 to 7.5% in 2019) but with significant age imbalance. Admissions peaked in January, whereas case fatality was highest during the summer months. Conclusions: These findings underscore the growing burden of CAP on healthcare systems, driven by an aging population and rising life expectancy. This has implications for both resource allocation and preventive strategies.

## 1. Introduction

Community-acquired pneumonia (CAP) remains one of the leading causes of morbidity and mortality worldwide, especially in the extremes of age [1], spanning both community and healthcare settings. CAP consistently ranks among the top causes of death globally, particularly affecting young children in low-resource settings and older adults in high-income countries. For adults, CAP incidence rises sharply with age, with the highest rates and case fatality observed in those over 65. Moreover, CAP is associated with significant health costs, primarily related to the need for primary care consultations, hospital admissions (both to the ward and to the intensive care unit [ICU]), and subsequent clinical deterioration after hospital discharge, which can last for several years [2]. In addition to the financial burden, it is important to acknowledge that CAP contributes to high antibiotic use, with future implications for the development of antibiotic resistance.

Major predisposing factors for CAP include advanced age, chronic comorbidities (such as chronic obstructive pulmonary disease (COPD), heart failure, diabetes, chronic hepatic, and kidney disease), and functional impairments affecting airway clearance (e.g., dysphagia, reduced consciousness) [3]. Lifestyle practices, especially poor oral hygiene, smoking, and alcohol use disorder, have also been linked to an increased risk of CAP [4]. Seasonal variation is well documented, with peak incidence occurring during winter months, correlating with cold temperatures and respiratory viral circulation [5].

In a cross-over Chinese study addressing more than 200,000 CAP deaths, non-optimum ambient temperature was shown to be related to the risk of mortality for various infectious causes. The impact of extremely low temperature on mortality of influenza-related pneumonia was 2.46, higher than for other viral CAP (1.89) or bacterial CAP (1.81). Extremely high temperatures were also related to an increased risk of death, but no significant difference was noted between agents [5].

Independently of severity, Streptococcus pneumoniae (pneumococcus) is the most frequently isolated pathogen [6], namely among patients with severe CAP, admitted to the ICU, and receiving invasive mechanical ventilation. Pneumococcal bacteremia is also common and has been associated with more severe disease [7]. Respiratory viruses (influenza, parainfluenza, respiratory syncytial virus) are more frequently recognized as etiological agents, either as a co-infection or as the primary pathogen [8,9].

Age is increasingly recognized as one of the strongest independent risk factors for both CAP incidence and mortality [10], and demographic aging is reshaping the disease burden in most high-income and middle-income countries [11].

Incidence of severe CAP as well as mortality climbs steeply from 40 years of age onward, with no differences between sexes [12]. In non-institutionalized elderly individuals, annual incidence ranges from 25 to 44 per 1000—roughly four times higher than that observed in those under 65 years [13]. Invasive pneumococcal disease shows the same gradient: rates rise from about 4–6 per 100,000 person-years at age 50–55 to 23–47 per 100,000 in those aged 80 and older, with some variation depending on the population studied [13]. Mortality follows an even sharper curve—adults aged 70 and older have the highest lower respiratory tract infection mortality globally (224.6 per 100,000), ahead of young children, historically the other high-risk extreme [12,13].

Aging-associated immunosenescence, as well as impaired antibody responses to polysaccharide and surface protein antigens, alongside a higher prevalence of comorbidities (especially COPD, heart failure, and diabetes), compound risk and worsen outcomes. This combination makes older adults simultaneously more susceptible to infection and more likely to have severe or complicated disease. Clinical presentation may also be more nonspecific and harder to detect, leading to substantial delays in treatment and worse outcomes [14].

Also, age-related swallowing dysfunction is common, reflecting age-associated reductions in muscle mass and connective tissue elasticity, limited mouth mobility, and neck stiffness [15].

Because the prevalence of elderly individuals in most populations is growing [11], absolute case counts and healthcare burden are expected to rise. This is already apparent in surveillance data: in the UK, clinically diagnosed pneumonia incidence in the ambulatory setting (primary care) increased from 1.50 to 2.22 per 1000 person-years between 2002 and 2017 [16], which was attributed mainly to population aging and improved case ascertainment. Regional studies in rapidly aging societies are now specifically tracking pneumonia burden as a marker of population health strain [17]. Data from Japan again showed an incidence rate that increased sharply with age, with people aged ≥85 years having a 10-fold higher incidence than those aged 15–64 years. Accordingly, the estimated annual number of adult CAP cases was 1,880,000, of whom 69.4% were aged ≥65 years.

Immunization programs have been proposed to reduce the burden and severity of CAP. Conjugate anti-pneumococcal broad-spectrum vaccines, influenza, and other respiratory virus (SARS-CoV-2, pertussis, respiratory syncytial virus) vaccination programs have been proposed to help prevent CAP burden [18]. However, a study in Spain addressing pneumococcal vaccination (with a relatively short-spectrum vaccine) failed to demonstrate a reduction in hospitalization of patients younger than 65 years [19], highlighting that direct health benefits may be more evident in high-risk patients, namely older or those with more comorbidities [20]. Nevertheless, additional benefit categories, such as productivity gains and healthcare cost savings (decline in visits to medical practitioners, inpatient stays, and prescription drugs), should also be considered when planning an immunization program [21].

In the US, age-adjusted CAP mortality may have declined substantially between 1999 and 2023 [20], although changes in disease labeling might have partly accounted for these trends. Nevertheless, pneumococcal and influenza vaccination have fundamentally altered the epidemiology of pneumococcal and viral pneumonia, though the picture is dynamic and still evolving. Reducing vaccine-type disease creates ecological space for non-vaccine serotypes and less common etiological agents.

Epidemiological studies in populations with CAP, particularly among those requiring hospital admission, including the identification of risk factors for in-hospital case fatality and the burden of health service utilization (especially the need for ICU admission and respiratory support), may help inform proper public health policies and continuously improve clinical outcomes.

Understanding seasonal variations of CAP incidence and hospitalization rate and its microbial etiology may also be important for establishing future CAP recommendations. CAP occurs throughout all seasons, albeit with substantial seasonal variations [22].

In this study, we evaluated CAP hospitalization data for adult patients in Portuguese mainland hospitals between 2000 and 2019. We address changes in population age, case fatality and mortality trends, rates of hospitalization, and seasonal variation.

## 2. Materials and Methods

The Central Administration of the Health System of the Portuguese Ministry of Health records administrative and clinical discharge data for all admissions to National Health System Hospitals, covering almost the whole resident population of mainland Portugal. This is an anonymized database, including aggregated details of all discharge diagnoses and clinical procedures performed on hospital inpatients, alive or deceased at discharge. All public hospitals are required to make this data available. Specially trained medical staff are responsible for data collection and introduction. The 9th Revision Clinical Modification of the International Classification of Diseases (ICD-9-CM) was used until 2016, subsequently transitioning to the 10th Revision Clinical Modification of the International Classification of Diseases (ICD-10-CM).

This study included all episodes of adult hospital admissions (>18 years) throughout 20 years, between 1 January 2000 and 31 December 2019. We computed administrative demographic data, namely age and sex, the presence of chronic comorbidities, and the month and year of admission. Case fatality at hospital discharge and hospital length of stay (LOS) were also computed.

The Central Administration of the Portuguese National Health System approved this study before giving access to the data, according to its Privacy and Personal Data Protection Policy (available at https://www.acss.min-saude.pt/2023/06/05/politica-de-privacidade-e-de-protecao-de-dados-pessoais, accessed on 20 July 2026). Since all protected individual patient information was inaccessible and only aggregated data were available, the requirement for patient informed consent was waived.

This study was performed in agreement with the Declaration of Helsinki statement of ethical principles for medical research and its subsequent amendments.

### 2.1. Community-Acquired Pneumonia

Hospitalization episodes of patients with a primary diagnosis of CAP were segregated for further analysis. Patients under 18 years of age, those for whom pneumonia was not the main diagnosis, immunocompromised patients, and those who did not have pneumonia “present on admission” were excluded.

The diagnosis of pneumonia was identified using the following codes: ICD-9-CM codes 480–486 and 487.0 and ICD-10-CM codes J12–J18, J10.0X, and J11.0X.

From 2013 onwards, this coding was supplemented with the “present on admission” code, added to the pneumonia diagnosis. Individuals with HIV infection (ICD-9-CM codes: 042-044; ICD-10-CM codes: B20) were excluded from the study, as well as those who were immunocompromised due to anti-cancer or immunosuppressive treatment (ICD-9-CM code: E933.1; V87.41 ICD-10-CM codes: D61.810, D70.1, Z92.21) and transplant recipients (ICD-9-CM codes: V42, except for corneal transplant status V42.5; ICD-10-CM codes: T86.XX and Z94.X, except for corneal transplant status Z94.7).

The general population data and the number of available hospital beds were obtained from the PORDATA portal (https://www.pordata.pt/db/portugal/ambiente+de+consulta/tabela accessed on 22 August 2026 and https://www.pordata.pt/portugal/hospitais+numero+e+camas-142, respectively accessed on 21 July 2026) and represent the estimates of the resident population, categorized by age and sex, for each year of the study.

### 2.2. Epidemiology

We used a descriptive statistical approach to derive the relevant indicators for this study. The study population was stratified into four age groups according to age at hospital admission: 18–40, 41–65, 66–80, and >80 years. For finer resolution among the oldest patients, those aged >75 years were further subdivided into four groups: 75–80, 81–85, 86–90, and >90 years.

Incidence rates of CAP requiring hospital admission were calculated per 1000 inhabitants by age group, month, and year of admission. We also examined temporal trends by comparing the absolute number of CAP hospitalization episodes with admission and case fatality rates over the 20-year study period. The absolute number (and percentage) of hospitalization episodes treated with invasive mechanical ventilation (IMV), according to patients’ age and year of admission, was calculated.

Age-standardized hospitalization rate, case fatality rate, and mortality rate were computed using a standardized European population.

The hospital LOS was computed for all hospitalization episodes with CAP for each year. Sub-analyses included different age groups, different months, and survivors. Incidence and case fatality of CAP requiring hospital admission were calculated according to the month of hospital admission.

The potential impact of a reduction in CAP requiring hospital admission incidence on hospital bed occupancy was estimated.

### 2.3. Statistical Analysis

Continuous variables were expressed as mean (standard deviation) and/or median (interquartile range) according to data distribution; the discrete variables were expressed as total number (percentage). Statistical significance analysis was performed using the chi-square test (for discrete variables), Student t-test, or Mann–Whitney U test (for continuous variables). A one-way ANOVA test was used for multiple comparisons. Correlations were evaluated with the Pearson correlation coefficient. A formal time trend analysis of CAP hospitalization, case fatality rates, and mortality was performed using an age-standardized negative binomial regression and a joinpoint (segmented) regression. Annual percent change with 95% confidence intervals was computed.

Statistical analysis was performed using IBM SPSS Statistics v.31.0 (IBM, Somers, NY, USA) and the Microsoft Excel spreadsheet (Microsoft Corp., Redmond, WA, USA). All statistics were two-tailed, and the significance level was set at *p* < 0.05.

## 3. Results

During the 20-year study period, there were 755,233 episodes of CAP admitted to Portuguese mainland hospitals (50.3% male patients). The mean age was 75.3 ± 15.2 years, slightly lower for males (74.9 ± 15.0 vs. 75.7 ± 15.4 years; Student *t*-test, *p* < 0.001). The median age was 79 years (69–86), and over 50% of patients were ≥80 years (Table 1).

Throughout the study period, in the hospitalization episodes of CAP, the mean age rose steadily, from 70.1 ± 17.0 years in 2000 to 77.7 ± 14.1 years in 2019 (ANOVA, *p* < 0.001).

The case fatality rate over these 20 years was 22.0%. Non-survivors were older (age 81.0 ± 11.1 vs. 73.7 ± 15.8; Student *t*-test, *p* < 0.001), and there was a strong correlation between lethality and older age (Table 1). The number of hospitalization episodes of CAP among patients older than 85 years increased progressively over the years, and these patients had a case fatality rate above 30% (Table 2). Notably, the hospitalization episodes of CAP among the population older than 90 years increased almost threefold over these 20 years, from 4.7% to 13.9% (Table 2). For the whole population, case fatality increased slightly during the study period, although a slight absolute decrease was observed from 2016 onwards.

### 3.1. Rate of Hospitalization

**Figure 1:**
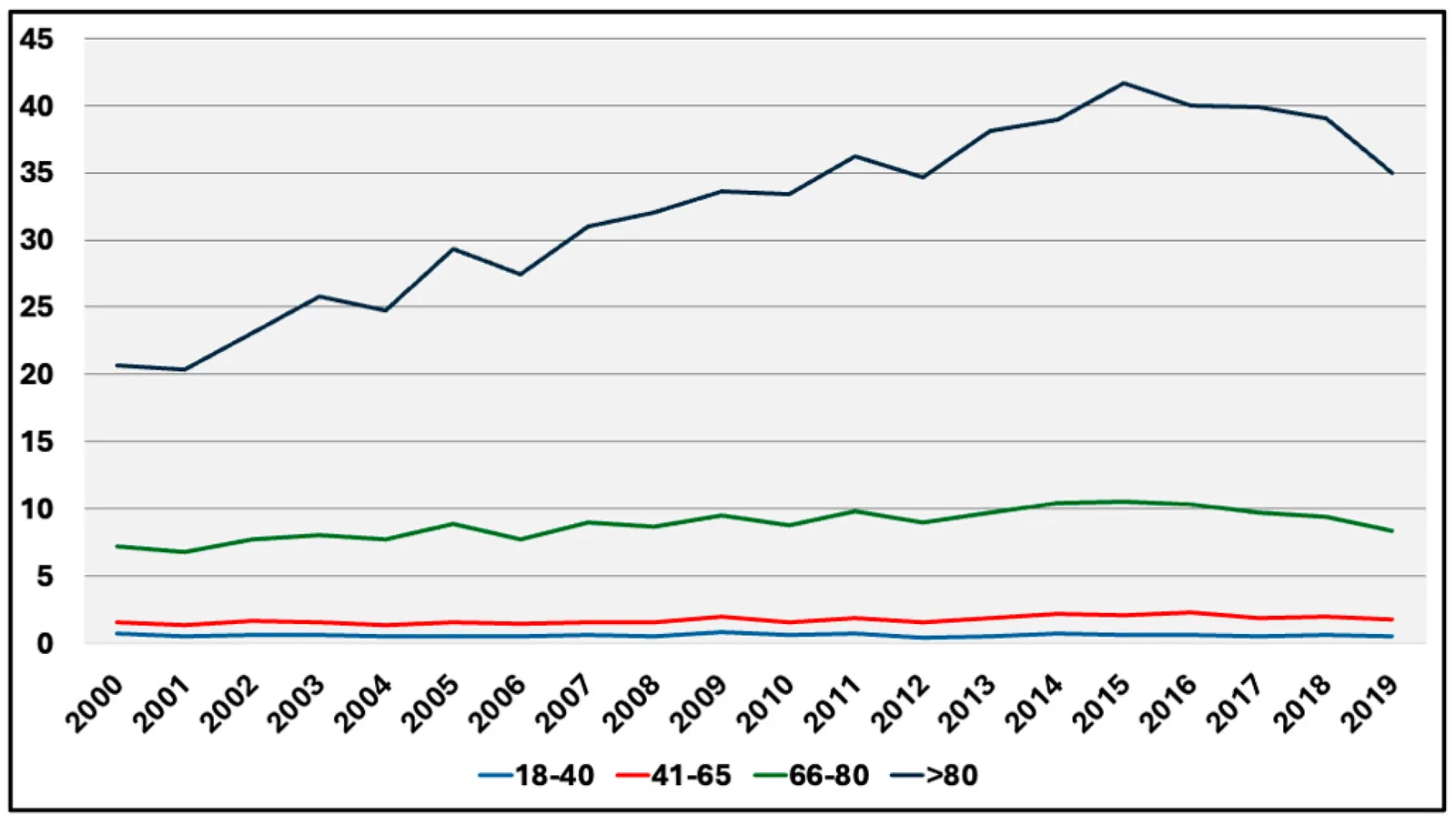
Annual rate of hospital admissions for community-acquired pneumonia per 1000 individuals by age group. Older patients had a higher admission rate during this period. From 2015 onwards, there was a slight decrease in hospital admissions.

During the studied period, the rate of hospitalization episodes was almost stable for all patients younger than 80 years (0.5–0.6 per 1000 persons-year for those younger than 40 years, 1.5–2.0 per 1000 persons-year for those aged 41–65 years, and 7.5–9.5 per 1000 persons-year for those aged 66–80 years). Even so, a slight decrease was noted for the whole population from 2015 onwards. Conversely, over the same time frame, a sharp increase was noted for those aged over 80 years, from 20.5 to 35 per 1000 persons-year (Figure 1), paralleling the increase in mean age of this sub-group (Table 2). Case fatality almost paralleled the population mean age (Pearson correlation coefficient, r = 0.95).

**Figure 2:**
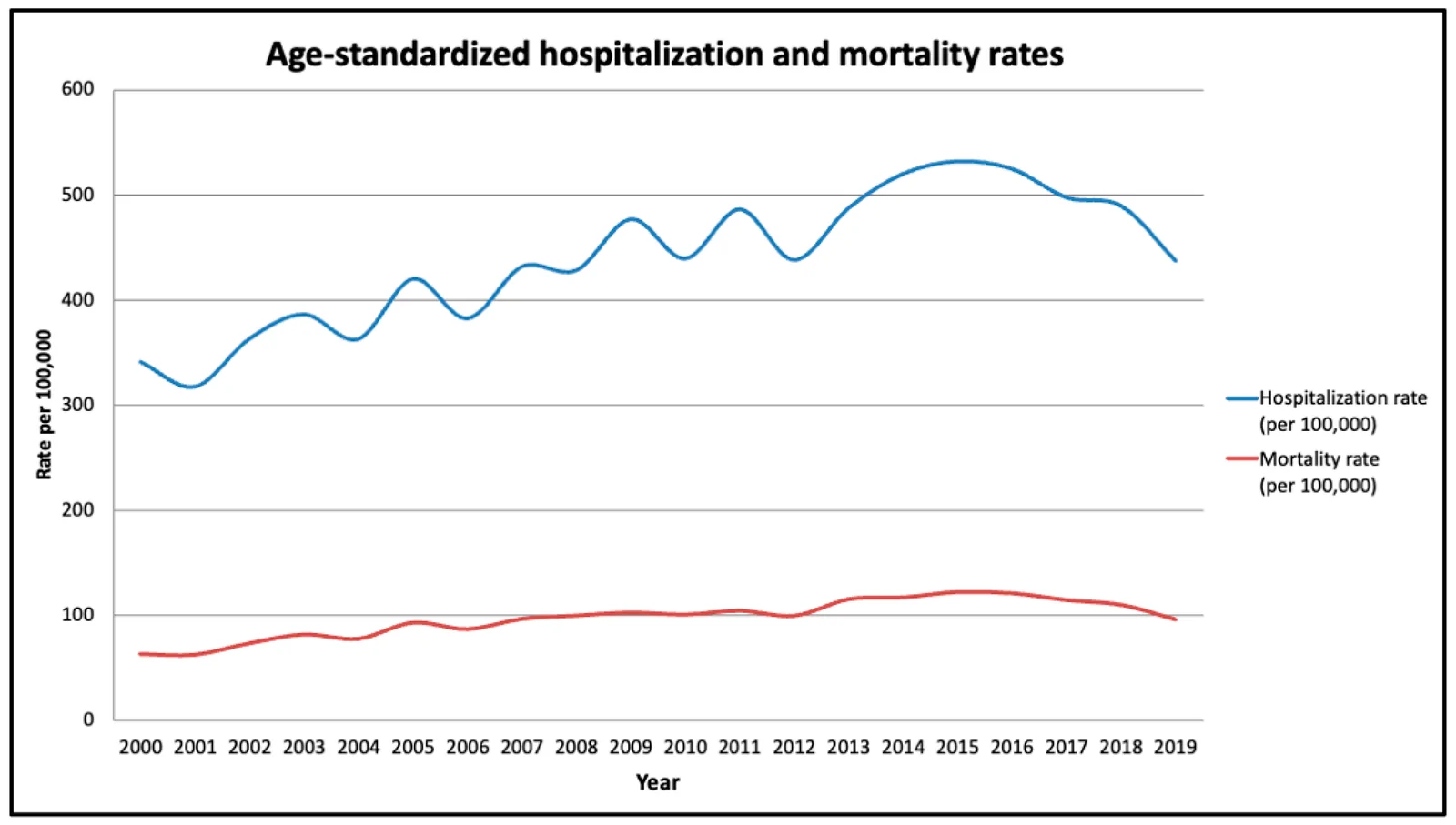
Age-standardized hospitalization and mortality rates during the 20-year period.

The age-standardized hospitalizations rate roughly climbed from 340 to a peak near 530 per 100,000 around 2015–2016, then eased off toward 2019. Case fatality rose steadily from near 17% (2000) to plateau around 22–24% from 2013 onward—meaning that, of those hospitalized, a growing share died, even after accounting for the aging population. Age-standardized mortality nearly doubled from near 63 to near 120 per 100,000 before also declining slightly at the end (Figure 2). 

### 3.2. Time Trend Analysis

In the age-standardized negative binomial regression, increasing trends for hospitalization and mortality rates were observed (1.1%/year [95% CI 0.9 to 1.4%], *p* < 0.001 and 3.1%/year [95%CI 2.7 to 3.4%], *p* < 0.001, respectively).

A joinpoint segmented regression was able to unmask a clear change in direction partway through the series in the year 2016: Hospitalization rate change from an upward trend of 1.7%/year (95% CI 1.4 to 2.1%) to −5.6%/year (95% CI −7.5 to −3.5%); likelihood-ratio test vs. single-trend model: χ^2^ = 36.8, *p* < 0.001. Mortality rate change from an upward trend of 3.9%/year (95% CI 3.5 to 4.2%) to −6.0% (95% CI −8.2 to −3.7%); likelihood-ratio test vs. single-trend model: χ^2^ = 49.3, *p* < 0.001.

The case fatality rate also had a joinpoint in 2006, preceding the hospitalization and mortality ones, from a 2.5%/year increase (95% CI 2.0 to 2.9%) to a plateau of 0.1%/year (95% CI −0.3% to 0.0%). This post-2016 decline may have been influenced by coding changes (from ICD-9-CM to ICD-10-CM).

Accordingly, the 2006–2016 period rise in mortality seems to be driven mainly by more people being hospitalized with CAP (and shifting age structure) and not by hospital care becoming less effective.

Of note, in 2016 there was a change in the codification system, from ICD-9-CM to ICD-10-CM. This could have influenced the changes in hospitalization and mortality rate described. A sensitivity analysis focusing on 2013 (when the “present on admission” criteria was introduced) also noted a significant change in trend, especially in the case fatality rate (which changed from a growing trend to a plateau afterwards). The influence of this change cannot be ruled out.

### 3.3. Hospital Length of Stay

The CAP hospitalization episodes had a median length of stay (LOS) of 9 days (IQR 5–14), which remained largely constant throughout the study period (ranging from 8 to 9 days). The median LOS was lower than the mean (11.7 days), indicating prolonged hospitalizations in a subset of episodes.

Non-survivors had a lower median hospital stay than survivors (6 (2–13) vs. 9 (6–14), Mann–Whitney U test, *p* < 0.001). The mode of non-survivors was 1 day (7 days for survivors), and 26.2% died within the first 48 h of hospital admission. This finding further reinforces the importance of a sepsis fast-track approach to these patients [23].

The median hospital LOS was similar between genders. Deceased older patients (≥66 years) had a significantly lower mean LOS than survivors (median 6 (2–13) vs. 9 (6–14) days, Mann–Whitney U test, *p* < 0.001), and this difference was less profound in deceased younger patients (≤65 years), median 7 (2–17) vs. 8 (5–13), Mann–Whitney U test, *p* < 0.001).

The mean number of hospital days per year of hospitalization episodes of CAP was 437,856 days, which approximately accounts for 6.4% of all hospital beds.

Reducing the number of CAP episodes in 2019 by just 5% would have resulted in approximately 28,915 fewer hospital days.

### 3.4. Invasive Mechanical Ventilation

**Figure 3:**
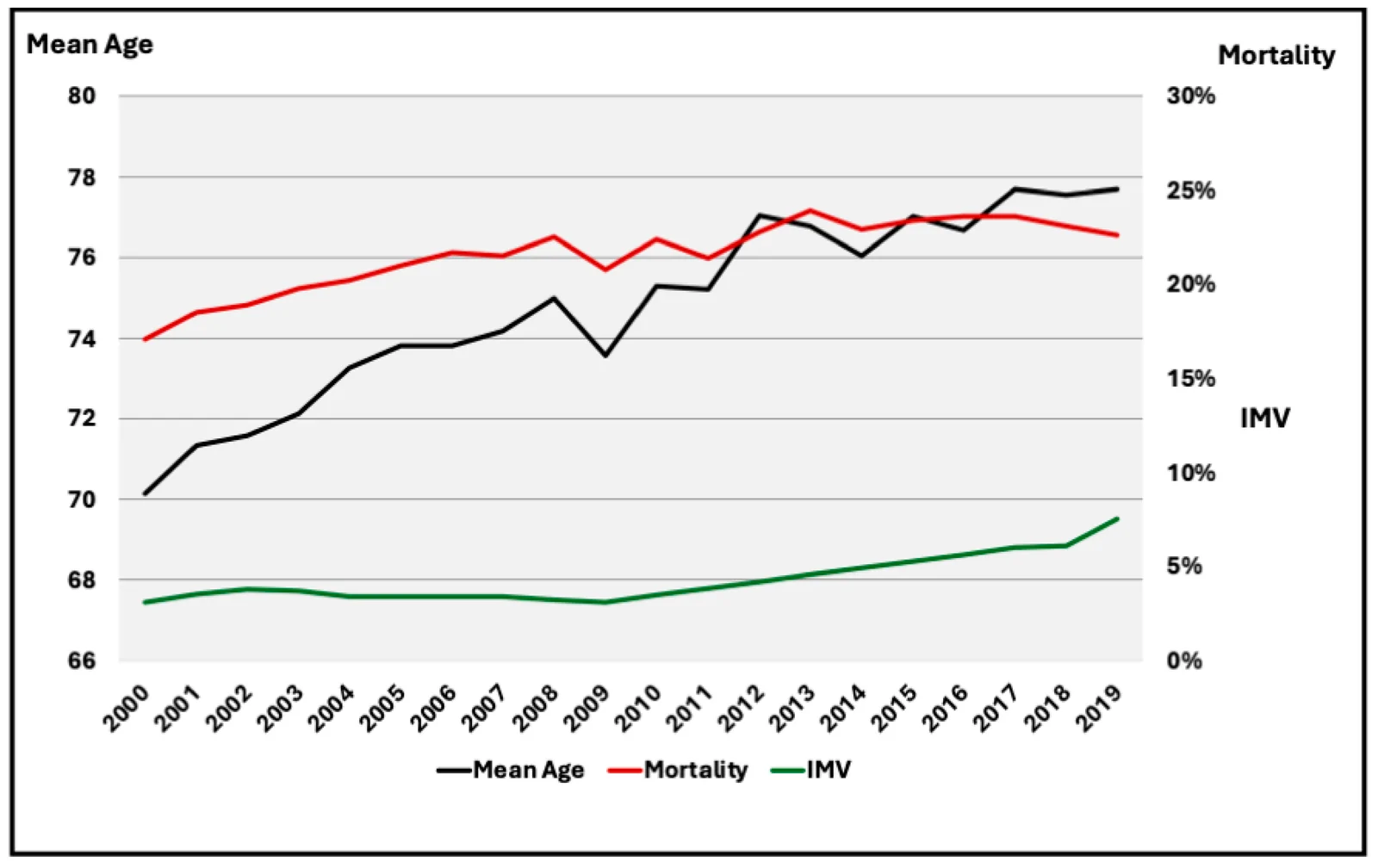
Yearly evolution of mean age, use of invasive mechanical ventilation, and mortality in hospitalization episodes for community-acquired pneumonia. A close relationship between mean age and case fatality was noted. Between 2010 and 2016, data for invasive mechanical ventilation were not available.

The proportion of hospitalization episodes of CAP where invasive mechanical ventilation (IMV) was used may have increased during the last years of the study, possibly reflecting an expansion in the number of ICU beds in Portugal (Figure 3). Notably, these data were unavailable between 2010 and 2016 (Table 1).

The rate of patients receiving IMV peaked in those aged between 41 and 65 years and decreased with age thereafter, although case fatality increased in older patients, possibly reflecting treatment limitations.

### 3.5. Seasonality

**Figure 4:**
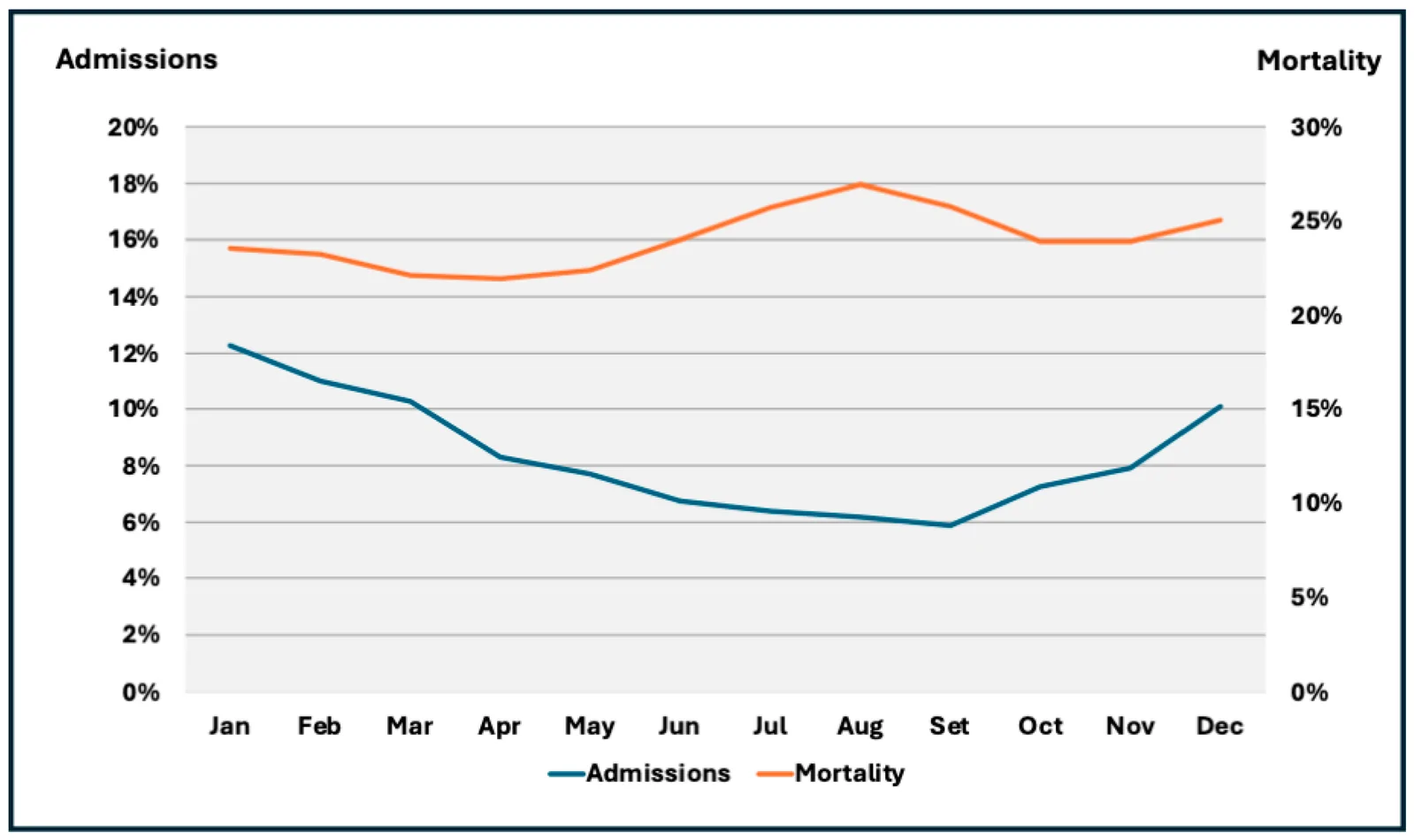
Hospital admissions for community-acquired pneumonia peaked during cold months, especially January (left bar); on the other hand, case fatality was maximum in the summer, peaking in August (right bar).

As expected, admissions were higher during the wintertime, peaking in January (12.2%), and reaching the lowest level in September (5.9%). However, a lethality peak was noted in summer, mainly from July to September (Figure 4), reaching over 25%. On the other hand, April had the lowest mortality (22.0%).

## 4. Discussion

This study addresses hospitalization episodes of patients admitted with CAP to Portuguese hospitals across 20 years. Throughout the study period, a demographic transition was noted, with a progressive rise in the mean age of our cohort, from 70.1 ± 17.0 to 77.7 ± 14.1 years, together with the near tripling of hospitalization episodes of patients older than 90 years (from 4.7% to 13.9%). This reflects the marked population aging happening in Portugal, with the proportion of residents older than 65 years exceeding 24%, and projections pointing to a continued rise in the old age ratio in the coming decade [24]. A steep rise among CAP hospitalizations of patients older than 80 in our cohort (from 20.5 to 35.0 per 1000 persons-year) mirrors internationally reported gradients in CAP hospitalization by age. A systematic review of population-based studies found hospitalization rates of 1830 per 100,000 adults aged ≥65 years compared with 199 per 100,000 in those younger than 65 [25]. Our data suggest this gradient has steepened further over two decades, in parallel with the growing share of “oldest-old” patients in the general population.

The age-standardized case fatality and mortality rates peaked around 2016, then slowly declined toward 2019. Increasingly higher mortality rates were noted in older age groups. This was consistent with an early study that reported an overall case fatality of 21.9% among hospitalized patients, rising from 7% in those aged ≤30 years to 38% in those aged 81–90 years [26]. More recent series confirm this age mortality gradient, with reported hospital case fatality for CAP ranging between 20% and 50% worldwide, consistently higher among older patients [27]. Accordingly, in our cohort, in the increasing number of hospitalization episodes of patients older than 85 years, the case fatality rate was consistently above 30%, and this may have influenced our results [26].

This pattern was consistent in our database: between 2000 and 2009, a hospital case fatality rate of 20.4% was noted, and deceased patients were older, averaging 79.8 years vs. 71.3 years among survivors [28]. In the second half of our cohort, case fatality again rose sharply with age, to over 40% in the oldest group [12]. This continuity of risk across the 20 years of our study is robust and provides strong evidence for this effect. Accordingly, old age remained a significant risk factor for severe CAP and mortality.

Two significant changes in the codification system were introduced during the study period (the “present on admission” code in 2013 and the change between ICD-9-CM and ICD-10-CM in 2016). In both these periods, we noted a significant trend change in hospitalization, case fatality, and mortality rates, and the effect of these codification changes cannot be ruled out.

Older patients may warrant IMV or admission to a higher level of care [29], and an increase in IMV in this sub-group was noted in our cohort. Nevertheless, severe CAP may also serve as a common pathway that precipitates functional decline or death in older patients, and the planning of realistic goals of care with the patient and the family is recommended to improve the overall satisfaction [30]. Antibiotic use in the context of end-of-life CAP is quite common, but patients seldom benefit, and development or selection of multidrug-resistant organisms may be facilitated [31].

Hospital LOS remained remarkably stable during the study period, and CAP patients occupied a mean of roughly 6.4% of all hospital beds. This is in keeping with reports from other CAP cohorts, where median stays consistently are in the range of 7–10 days [32], although a declining pattern has also been reported [33], probably related to organizational issues. More clinically relevant is the fact that 26.2% of deaths occurred within 48 h of admission, which is consistent with the recognized potential for CAP to progress to fulminant respiratory failure and early death irrespective of antimicrobial therapy. This is particularly relevant in patients with comorbidities and impaired physiological reserve [34] and reinforces the need for early risk stratification and sepsis “fast-track” pathways to identify patients at risk of rapid deterioration early in the emergency room [35].

The proportion of hospitalization episodes of patients receiving invasive mechanical ventilation rose in the last years of our study, possibly related to the substantial expansion of Portuguese intensive care capacity over this period. Portugal entered the 2000s with one of the lowest ICU bed ratios in Europe (4.2 beds per 100,000 inhabitants) [36,37], but this ratio has since increased considerably, reaching approximately 6.5 per 100,000 around 2020 and peaking near 8.8 per 100,000 in 2022 before stabilizing close to 8.0 [36]. Accordingly, IMV was reported in a growing number of hospitalization episodes of CAP patients, rising from 3.1% in 2000 to 7.5% in 2019, but with significant age imbalance (Table 2). The same increasing trend was noted in the USA, between 1993 and 2009, with a concomitant decrease in mortality (OR per 5 years, 0.89; 95% CI, 0.88–0.90) [38], which we did not find in our cohort.

### 4.1. Seasonality of Admissions and Case Fatality

A dissociation between admission volume, which peaked in winter (January), and case fatality, which peaked in summer (July–September), has been previously described [39]. A large Spanish study of hospitalizations for respiratory infections or respiratory failure found that while admissions were consistently higher during the cold season, in-hospital case fatality followed the opposite pattern, peaking during the warmest months and correlating with high ambient temperature [40]. This seasonality has been attributed to a combination of factors: winter admissions are driven by a large volume of relatively typical, viral infections, whereas summer admissions may represent a smaller but more physiologically vulnerable subset of patients [41], compounded by direct heat-related physiological stress in an aging, frail population, potentially more important in a time of climate change [42]. Complications related to heat and a delay in identifying respiratory infections (particularly when patients do not present with classic symptoms) may lead to worse outcomes. This heat-associated risk may even replace the risk of death due to cold temperatures [43].

Due to the limitations of our database, we could not perform a multivariable analysis, and our findings must be addressed as exploratory. Future prospective studies should investigate this point further.

### 4.2. Prevention of Community-Acquired Pneumonia

Despite the high burden of CAP incidence and mortality, which could be over four times higher than that of myocardial infarction or stroke, the economic effort for preventive strategies for CAP constitutes less than 10% of the investment allocated to the prevention of these other diseases [44].

According to our data, efforts to prevent CAP should prioritize older patients. Notably, CAP in very elderly patients has distinct clinical and prognostic implications. Immunosenescence, multimorbidity, frailty, and polypharmacy increase susceptibility to infection and worsen outcomes [45]. In this population, CAP is often aspiration-related and should be addressed as such [46]. Evaluation and prevention of swallowing dysfunction may help prevent microaspiration and CAP [47,48]. In addition to improving nutrition and physical activity, identifying and slowing the progression of frailty and avoiding polypharmacy [45] may all help to reduce CAP in this at-risk group. Furthermore, simple measures that may reduce the incidence of CAP, such as ambulation and mouth hygiene, may even improve the quality of life and are commonly missed.

Immunization may help to decrease the incidence and severity of CAP. In 2015, the Portuguese health authorities recommended the 13-valent and the 23-valent pneumococcal conjugate vaccines to all patients older than 65 years. Pneumococcal immunization, along with a sustained increase of over 70%, in influenza virus vaccination coverage rate, especially in high-risk and older patients [49], may have contributed to a decrease in CAP mortality. The benefit of anti-pneumococcal vaccination for the prevention of severe CAP is well known [50], and a 23% reduction in the incidence of invasive pneumococcal disease has been described [51]. In addition to this, several studies demonstrate the impact of pediatric vaccination on the adult population, particularly in older individuals, specifically the benefits in reducing the burden of disease caused by Streptococcus pneumoniae, one of the main microorganisms of CAP [52,53,54].

### 4.3. Limitations

This study has several limitations, particularly related to the administrative nature of the official databases used. First, due to the study design, it was not possible to confirm the accuracy of CAP diagnosis. Second, no microbiological data were obtained. Third, some cases of nosocomial pneumonia may have been included in this analysis due to coding errors, as well as the inclusion of end-of-life pneumonia due to hospital accessibility and the reluctance of families to let patients die at home without medical support. Fourth, the database does not allow determining the vaccination status of the admitted individuals. Fifth, the impact of other known risk factors, such as smoking, alcoholism, and comorbidities like COPD and other respiratory diseases; cardiovascular diseases; and diabetes mellitus were not evaluated. Sixth, we included all episodes of CAP admitted to the hospital and some patients may have been included more than once, which affects the calculation of mortality rates. Seventh, we did not have data on patients who might have died in the ambulatory setting. Finally, due to changes in the reporting codification during the study period, some data may have been missed (like the IMV between 2010 and 2016). Moreover, the changing trends of hospitalization and mortality rates may have been influenced by the introduction of the “present on admission” in 2013 and the change from the ICD-9-CM to ICD-10-CM codification system in 2016.

This study also has some strengths: it covers a long (20 years) continuous period, includes almost all hospitals in mainland Portugal, and the reporting of data to the national database is mandatory.

## 5. Conclusions

The epidemiology of hospitalized CAP, both the hospitalization rate and mortality, seems to be dependent on age mix. This finding underscores the growing burden of CAP on healthcare systems, driven by an aging population and rising life expectancy. General preventive strategies may help to improve CAP outcomes. Even a small reduction in the rate of hospitalized CAP may have a significant impact on the use of healthcare resources. Public health interventions should be tailored according to population demographics.

## Figures and Tables

**Table 1:** Yearly hospital admissions with community-acquired pneumonia according to age group.

Age:	18–40			41–65			66–80			>80		
Year	*N* (%)	IMV	Case Fatality	*N* (%)	IMV	Case Fatality	*N* (%)	IMV	Case Fatality	*N* (%)	IMV	Case Fatality
2000	2052 (8.7%)	3.3%	3.4%	4951 (20.9%)	5.3%	7.9%	9470 (40.0%)	3.6%	17.6%	7206 (30.4%)	1.0%	26.7%
2001	1681 (7.5%)	4.9%	4.0%	4297 (19.1%)	6.0%	9.0%	9024 (40.2%)	3.8%	18.5%	7440 (33.2%)	1.4%	27.2%
2002	1769 (6.8%)	5.3%	3.7%	5108 (19.6%)	6.2%	8.1%	10,505 (40.3%)	4.2%	17.7%	8711 (33.4%)	1.5%	29.7%
2003	1963 (7.0%)	5.4%	3.9%	5031 (18.0%)	6.4%	9.1%	10,980 (39.2%)	4.2%	18.8%	10,004 (35.8%)	1.3%	29.5%
2004	1441 (5.4%)	4.0%	3.9%	4504 (16.9%)	6.0%	8.9%	10,701 (40.3%)	4.0%	18.6%	9936 (37.4%)	1.5%	29.3%
2005	1612 (5.1%)	4.7%	4.6%	5146 (16.4%)	6.3%	9.1%	12,295 (39.3%)	3.9%	19.2%	12,249 (39.1%)	1.4%	29.9%
2006	1625 (5.6%)	4.3%	4.5%	4746 (!6.3%)	7.1%	9.9%	10,857 (37.3%)	3.9%	20.3%	11,903 (40.9%)	1.4%	30.0%
2007	1737 (5.2%)	4.7%	4.4%	5370 (15.9%)	6.5%	9.6%	12,625 (37.4%)	4.2%	18.6%	13,994 (41.5%)	1.4%	30.6%
2008	1518 (4.5%)	4.9%	3.8%	5280 (15.5%)	6.5%	10.5%	12,312 (36.1%)	3.7%	19.8%	14,973 (43.9%)	1.5%	30.7%
2009	2236 (5.7%)	6.4%	3.6%	7058 (18.1%)	5.5%	8.6%	13,498 (34.6%)	3.5%	18.9%	16,219 (41.6%)	1.2%	30.0%
2010	1667 (4.5%)		4.0%	5622 (15.3%)		10.6%	12,731 (34.5%)		19.7%	16,845 (45.7%)		30.3%
2011	2019 (4.7%)		4.6%	6716 (15.8%)		10.2%	14,271 (33.5%)		18.9%	19,589 (46.0%)		28.7%
2012	1194 (3.0%)		4.6%	5432 (13.7%)		10.4%	13,112 (33.1%)		19.4%	19,897 (50.2%)		29.6%
2013	1353 (3.0%)		4.9%	6749 (15.0%)		13.2%	14,369 (31.9%)		21.0%	22,532 (50.1%)		30.0%
2014	1823 (3.7%)		5.4%	7823 (16.0%)		11.5%	15,534 (31.7%)		20.2%	23,750 (48.5%)		29.7%
2015	1571 (3.1%)		5.2%	7450 (14.5%)		12.7%	16,037 (31.3%)		19.9%	26,152 (51.1%)		29.7%
2016	1546 (3.0%)		4.3%	8177 (15.9%)		12.9%	15,883 (30.9%)		20.3%	25,777 (50.2%)		30.2%
2017	1218 (2.4%)	6.5%	5.1%	7043 (14.1%)	7.5%	13.2%	15,241 (30.6%)	6.9%	20.0%	26,361 (52.9%)	5.0%	29.4%
2018	1351 (2.7%)	6.6%	4.1%	7247 (14.5%)	8.5%	12.6%	14,946 (29.9%)	7.3%	19.9%	26,465 (52.9%)	4.8%	28.8%
2019	1123 (2.5%)	5.7%	5.6%	6679 (14.6%)	9.6%	13.3%	13,622 (29.8%)	8.9%	19.8%	24,289 (53.1%)	6.3%	27.5%

Number of admissions for community-acquired pneumonia, hospital case fatality rate (%), and rate of the use of IMV (invasive mechanical ventilation). Between 2010 and 2016, data on IMV were not available.

**Table 2:** Yearly hospital admissions with community-acquired pneumonia of older patients.

Age:	76–80			81–85			86–90			>90		
Year	*N* (%)	IMV	Case Fatality	*N* (%)	IMV	Case Fatality	*N* (%)	IMV	Case Fatality	*N* (%)	IMV	Case Fatality
2000	3920 (16.6%)	3.4%	20.4%	3514 (14.8%)	1.3%	23.4%	2587 (10.9%)	0.8%	28.6%	1105 (4.7%)	0.4%	32.2%
2001	3834 (17.1%)	2.7%	21.4%	3478 (15.5%)	1.8%	25.2%	2700 (12.0%)	1.3%	28.0%	1262 (5.6%)	0.3%	30.7%
2002	4486 (17.2%)	3.5%	20.7%	4129 (15.8%)	2.0%	26.6%	3102 (11.9%)	1.2%	29.6%	1480 (5.7%)	0.8%	38.4%
2003	4768 (17.0%)	3.5%	21.8%	4678 (16.7%)	1.7%	25.9%	3536 (12.6%)	1.0%	30.5%	1790 (6.4%)	0.6%	37.0%
2004	4819 (18.1%)	3.4%	22.0%	4754 (17.9%)	2.1%	26.4%	3398 (12.8%)	1.1%	30.6%	1784 (6.7%)	0.7%	34.6%
2005	5622 (18.0%)	2.9%	21.9%	5879 (18.8%)	2.0%	26.9%	3997 (12.8%)	1.0%	30.0%	2373 (7.6%)	0.8%	37.1%
2006	4900 (16.8%)	3.0%	22.9%	5661 (19.4%)	2.1%	26.7%	4007 (13.8%)	1.1%	31.1%	2235 (7.7%)	0.4%	36.4%
2007	5795 (17.2%)	3.3%	21.4%	6676 (19.8%)	2.0%	27.3%	4680 (13.9%)	1.0%	31.2%	2638 (7.8%)	0.5%	38.1%
2008	5832 (17.1%)	2.9%	22.5%	7028 (20.6%)	2.1%	26.9%	5023 (14.7%)	1.3%	32.1%	2912 (8.5%)	0.4%	37.6%
2009	6278 (16.1%)	3.0%	22.0%	7477 (19.2%)	1.9%	25.9%	5623 (14.4%)	0.8%	31.2%	3119 (8.0%)	0.2%	37.9%
2010	6103 (16.6%)		22.6%	7557 (20.5%)		26.1%	6016 (16.3%)		31.1%	3272 (8.9%)		38.4%
2011	6929 (16.3%)		21.7%	8541 (20.1%)		25.2%	7113 (16.7%)		29.7%	3935 (9.2%)		34.5%
2012	6543 (16.5%)		22.6%	8370 (21.1%)		25.8%	7502 (18.9%)		30.4%	4025 (10.2%)		36.2%
2013	7086 (15.7%)		22.6%	9302 (20.7%)		26.7%	8390 (18.6%)		30.3%	4840 (10.8%)		35.9%
2014	7510 (15.3%)		21.9%	9730 (19.9%)		25.8%	8795 (18.0%)		30.0%	5225 (10.7%)		36.5%
2015	8017 (15.7%)		21.8%	10,530 (20.6%)		25.8%	9591 (18.7%)		29.9%	6031 (11.8%)		36.3%
2016	7647 (14.9%)		22.7%	9990 (19.4%)		26.2%	9504 (18.5%)		29.9%	6283 (12.2%)		37.2%
2017	7211 (14.5%)	6.4%	21.0%	10,152 (20.4%)	6.1%	25.3%	9700 (19.5%)	4.9%	29.4%	6509 (13.1%)	3.4%	35.7%
2018	6864 (13.7%)	6.7%	21.1%	10,143 (20.3%)	5.8%	24.9%	9583 (19.2%)	4.7%	28.2%	6739 (13.5%)	4.9%	35.7%
2019	6124 (13.4%)	8.7%	21.6%	8951 (19.6%)	7.1%	23.5%	9006 (19.7%)	6.5%	26.9%	6332 (13.9%)	5.6%	34.1%

Number of admissions for community-acquired pneumonia, hospital case fatality rate (%), and rate of the use of IMV (invasive mechanical ventilation). Between 2010 and 2016, data on IMV were not available.

## Data Availability

The datasets supporting this study’s findings are available from the Central Administration of the Portuguese National Health System. Still, restrictions apply to their availability, according to their Privacy and Personal Data Protection Policy. Access to raw data can be requested from Cláudia Borges (cborges@acss.min-saude.pt), Luís Faustino (lfaustino@acss.min-saude.pt), and Vanessa Silva (vcsilva@acss.min-saude.pt). H.O. act as the guardian of the raw data used in this study. After permission from the Central Administration of the Portuguese National Health System, authors can provide the data used in this study upon reasonable request.

## Abbreviations

The following abbreviations are used in this manuscript:

CAP	Community-acquired pneumonia
ICU	Intensive care unit
COPD	Chronic obstructive pulmonary disease
ICD-9-CM	9th Revision Clinical Modification of the International Classification of Diseases
ICD-10-CM	10th Revision Clinical Modification of the International Classification of Diseases
LOS	Length of stay
IMV	Invasive mechanical ventilation
OR	Odds ratio
CI	Confidence interval
